# Supplementary material for: Lifestyle Risk Factors for Overweight/Obesity in Spanish Children
Source: Children (Basel). 2022 Dec 12;9(12):1947. doi: 10.3390/children9121947 (PMC9777298; doi:10.3390/children9121947)
Supplement: Supplementary file 1 [file children-09-01947-s001.zip › children-2043054-supplementary.pdf]

ALIMENTANDO EL CAMBIO

Centro	Participante	Fecha actual
		Día Mes Año

Cuestionario de Frecuencia de alimentos para Niños Pequeños

Información acerca de su hijo/a

Nos gustaría que nos describiese los hábitos de consumo de líquidos de su hijo en los últimos 1-2 meses.
Por lo tanto, es importante que éste cuestionario sea completado por la que persona que pasa más tiempo con el niño (a parte del tiempo en el colegio). Éste debe incluir todas las bebidas consumidas durante éste período. Debe incluir, así mismo, cualquier bebida que su hijo consuma fuera de casa, incluyendo la guardería, los restaurantes

Ejemplo: Si tu hijo/a toma tres tazas de chocolate con leche por semana, la respuesta debería ser:

**Grupos de alimentos**	**¿Con qué** frecuencia consume su hijo los siguientes productos?	Y ¿cúal es la **cantidad media** por día?	**Ejemplo** de tamaño de porciones	**Elija** la opción más frecuente
Leche azúcarada o chocolateada (Asturiana, cola cao)	o nunca o menos de una vez al mes<br>o 1-3 días por mes<br>o 1 día por semana<br>**x** 2-4 días por semana<br>o 5-6 días por semana<br>o todos los días	o 100 ml o menos<br>o entre 100 y 200 ml<br>**x** entre 200 y 300 ml<br>o entre 300 y 400 ml<br>o entre 400 y 500 ml<br>o entre 500 y 600 ml<br>o entre 600 y 700 ml<br>o entre 700 y 800 ml<br>o entre 800 y 900 ml<br>o entre 900 y 1000ml<br>o 1000 ml o más	*1 taza = 225 ml*<br>*1 lata= 200ml*<br>*1 botella pequeña de plástico= 300ml*<br>*1 botella grande de plástico= 500ml*<br><br>*Para tamaños de vaso, taza y cartón mire el apéndice.*	

ALIMENTANDO EL CAMBIO

Centro		Participante			Fecha actual Día		Mes		Año	

**Grupos de alimentos**	**¿Con qué** frecuencia consume su hijo los siguientes productos?	Y ¿cúal es la **cantidad media** por día?	**Ejemplo** de tamaño de porciones	**Elija** la opción más frecuente
Agua natural	o nunca o menos de una vez al mes<br>o 1-3 días al mes<br>o 1 día a la semana<br>o 2-4 días a la semana<br>o 5-6 días a la semana<br>o todos los días	o 100 ml o menos<br>o entre 100 y 200 ml<br>o entre 200 y 300 ml<br>o entre 300 y 400 ml<br>o entre 400 y 500 ml<br>o entre 500 y 600 ml<br>o entre 600 y 700 ml<br>o entre 700 y 800 ml<br>o entre 800 y 900 ml<br>o entre 900 y 1000 ml<br>o 1000 ml o más	*1 taza = 225 ml*<br>*1 botella pequeña de plástico= 500ml*<br><br>*Para tamaños de vasos y tazas, por favor, vea el apéndice*	
Bebidas azúcaradas<br><br>*(Coca Cola, Pepsi, Fanta, Sprite, Nestea)*	o nunca o menos de una vez al mes<br>o 1-3 días al mes<br>o 1 día a la semana<br>o 2-4 días a la semana<br>o 5-6 días a la semana<br>o todos los días	o 100 ml o menos<br>o entre 100 y 200 ml<br>o entre 200 y 300 ml<br>o entre 300 y 400 ml<br>o entre 400 y 500 ml<br>o entre 500 y 600 ml<br>o entre 600 y 700 ml<br>o entre 700 y 800 ml<br>o entre 800 y 900 ml<br>o entre 900 y 1000 ml<br>o 1000 ml o más	*1 taza = 225 ml*<br>*1 lata= 330ml*<br>*1 botella pequeña de plástico= 500ml*<br><br>*Para tamaños de vasos y tazas, por favor, vea el apéndice*	
Bebidas light<br><br>*(Cola light, Coca cola zero, Pepsi max, Fanta zero)*	o nunca o menos de una vez al mes mes<br>o 1-3 días al mes<br>o 1 día a la semana<br>o 2-4 días a la semana<br>o 5-6 días a la semana<br>o todos los días	o 100 ml o menos<br>o entre 100 y 200 ml<br>o entre 200 y 300 ml<br>o entre 300 y 400 ml<br>o entre 400 y 500 ml<br>o entre 500 y 600 ml<br>o entre 600 y 700 ml<br>o entre 700 y 800 ml<br>o entre 800 y 900 ml<br>o entre 900 y 1000 ml<br>o 1000 ml o más	*1 taza = 225 ml*<br>*1 lata= 330ml*<br>*1 botella = 500ml*<br><br>*Para tamaños de vasos y tazas, por favor, vea el apéndice*	

ALIMENTANDO EL CAMBIO

Centro	Participante	Fecha actual
		Día / Mes / Año

**Grupos de alimentos**	**¿Con qué** frecuencia consume su hijo los siguientes productos?	Y ¿cúal es la **cantidad media** por día?	**Ejemplo** de tamaño de porciones	**Elija** la opción más frecuente
Zumo de frutas natural recién exprimido	o nunca o menos de una vez al mes<br>o 1-3 días al mes<br>o 1 día a la semana<br>o 2-4 días a la semana<br>o 5-6 días a la semana<br>o todos los días	o 100 ml o menos<br>o entre 100 y 200 ml<br>o entre 200 y 300 ml<br>o entre 300 y 400 ml<br>o entre 400 y 500 ml<br>o entre 500 y 600 ml<br>o entre 600 y 700 ml<br>o entre 700 y 800 ml<br>o entre 800 y 900 ml<br>o entre 900 y 1000 ml<br>o 1000 ml o más	*1 taza= 225 ml*<br><br>*Para tamaños de vasos y tazas, por favor, vea el apéndice*	
Zumo de fruta industrial de brick/embotellado.<br>(*Don Simon, Juver/Disfruta, marcas blancas, etc)*	o nunca o menos de una vez al mes<br>o 1-3 días al mes<br>o 1 día a la semana<br>o 2-4 días a la semana<br>o 5-6 días a la semana<br>o todos los días	o 100 ml o menos<br>o entre 100 y 200 ml<br>o entre 200 y 300 ml<br>o entre 300 y 400 ml<br>o entre 400 y 500 ml<br>o entre 500 y 600 ml<br>o entre 600 y 700 ml<br>o entre 700 y 800 ml<br>o entre 800 y 900 ml<br>o entre 900 y 1000 ml<br>o 1000 ml o más	*1 taza = 225 ml*<br>*1 botella pequeña de plástico= 500ml*<br>*1 carton= 1l*<br><br>*Para tamaños de vasos y tazas, por favor, vea el apéndice*	
Té<br><br>(infusión, té negro, té verde, manzanilla, etc)	o nunca o menos de una vez al mes<br>o 1-3 días al mes<br>o 1 día a la semana<br>o 2-4 días a la semana<br>o 5-6 días a la semana<br>o todos los días	o 100 ml o menos<br>o entre 100 y 200 ml<br>o entre 200 y 300 ml<br>o entre 300 y 400 ml<br>o entre 400 y 500 ml<br>o entre 500 y 600 ml<br>o entre 600 y 700 ml<br>o entre 700 y 800 ml<br>o entre 800 y 900 ml<br>o entre 900 y 1000 ml<br>o 1000 ml o más	*1 taza = 225 ml*<br><br>*Para tamaños de vasos y tazas, por favor, vea el apéndice*	o con azúcar<br>o sin azúcar

ALIMENTANDO EL CAMBIO

Centro	Participante	Fecha actual Día / Mes / Año

**Grupos de alimentos**	**¿Con qué** frecuencia consume su hijo los siguientes productos?	Y ¿cúal es la **cantidad media** por día?	**Ejemplo** de tamaño de porciones	**Elija** la opción más frecuente
Batidos (todos los tipos)<br><br>(*Solo Fruta (Hero), FruitSimply (Sunnydelight)), etc*	o nunca o menos de una vez al mes<br>o 1-3 días al mes<br>o 1 día a la semana<br>o 2-4 días a la semana<br>o 5-6 días a la semana<br>o todos los días	o 100 ml o menos<br>o entre 100 y 200 ml<br>o entre 200 y 300 ml<br>o entre 300 y 400 ml<br>o entre 400 y 500 ml<br>o entre 500 y 600 ml<br>o entre 600 y 700 ml<br>o entre 700 y 800 ml<br>o entre 800 y 900 ml<br>o entre 900 y 1000 ml<br>o 1000 ml o más	*1 taza = 225 ml*<br>*1 lata= 330ml*<br>*1 carton=*<br>*1 botella de plástico pequeña= 500ml*<br><br>*Para tamaños de vasos y tazas, por favor, vea el apéndice*	
Leche	o nunca o menos de una vez al mes<br>o 1-3 días al mes<br>o 1 día a la semana<br>o 2-4 días a la semana<br>o 5-6 días a la semana<br>o todos los días	o 100 ml o menos<br>o entre 100 y 200 ml<br>o entre 200 y 300 ml<br>o entre 300 y 400 ml<br>o entre 400 y 500 ml<br>o entre 500 y 600 ml<br>o entre 600 y 700 ml<br>o entre 700 y 800 ml<br>o entre 800 y 900 ml<br>o entre 900 y 1000 ml<br>o 1000 ml o más	*1 taza = 225 ml*<br>*1 botella de plástico pequeña= 500ml*<br><br>*Para tamaños de vasos y tazas, por favor, vea el apéndice*	o Leche entera<br>o Semidesnatada<br>o Desnatada<br>o Fortificada/enriquecida (por ejemplo, con calcio, hierro, vitaminas)
Leche chocolateada o azucarada<br><br>(*Puleva, Central Lechera Asturiana, marcas blancas*)	o nunca o menos de una vez al mes<br>o 1-3 días al mes<br>o 1 día a la semana<br>o 2-4 días a la semana<br>o 5-6 días a la semana<br>o todos los días	o 100 ml o menos<br>o entre 100 y 200 ml<br>o entre 200 y 300 ml<br>o entre 300 y 400 ml<br>o entre 400 y 500 ml<br>o entre 500 y 600 ml<br>o entre 600 y 700 ml<br>o entre 700 y 800 ml<br>o entre 800 y 900 ml<br>o entre 900 y 1000 ml<br>o 1000 ml o más	*1 taza = 225 ml*<br>*1 lata= 200ml*<br>*1 botella pequeña de plástico= 300ml*<br>*1 botella grande de plástico= 500ml*<br><br>*Para tamaños de vasos y tazas, por favor, vea el apéndice*	

ALIMENTANDO EL CAMBIO

Centro	Participante	Fecha actual
		Día / Mes / Año

**Grupos de alimentos**	**¿Con qué** frecuencia consume su hijo los siguientes productos?	Y ¿cúal es la **cantidad media** por día?	**Ejemplo** de tamaño de porciones	**Elija** la opción más frecuente
Yogurt natural<br>*(sin azúcar añadido, yogur con edulcorante artificial)*<br><br>(*Danone, Danonino, Petit-suisse, marcas blancas*)	o nunca o menos de una vez al mes<br>o 1-3 días al mes<br>o 1 día a la semana<br>o 2-4 días a la semana<br>o 5-6 días a la semana<br>o todos los días	o 65 g o menos<br>o entre 65 y 195 g<br>o 195 g o más	*1 taza=125 mg*<br>*1 petit-suisse= 50 g*<br><br>*Para tamaños de vasos y tazas, por favor, vea el apéndice*	o Yogurt griego<br>oEntero<br>oSemidesnatado<br>oDesnatado<br>oFortificado/enriquecido (por ejemplo, con vitaminas calcio, hierro, vitaminas)<br>o Yogurt para niños
Yogurt de frutas aromatizado o azucarado<br>(*yogurt de frutas, yogurt con azúcar añadido por el consumidor…)*<br>*Leches fermentadas tipo Actimel…*<br>(*Danone, Danonino, Petit-suisse, marcas blancas*)	o nunca o menos de una vez al mes<br>o 1-3 días al mes<br>o 1 día a la semana<br>o 2-4 días a la semana<br>o 5-6 días a la semana<br>o todos los días	o 65 g o menos<br>o entre 65 y 195 g<br>o 195 g o más	*1 taza=125 mg*<br>*1 petit-suisse= 50 g*<br><br>*Para tamaños de vasos y tazas, por favor, vea el apéndice*	oEntera<br>oSemidesnatada<br>oDesnatada<br>oFortificada/ enriquecida (por ejemplo, con vitaminas, calcio, hierro)<br>○ Yogurt para niños

¡Muchas gracias por haber contestado a estas preguntas!

Por favor, compruebe que no se ha dejado ninguna pregunta por responder.

Por favor, anote la fecha en la que terminó de rellenar el cuestionario:

Día: __ __ __ __ __ __ __ __

ALIMENTANDO EL CAMBIO

Centro	Participante	Fecha actual: Día	Mes	Año

CUESTIONARIO PARA VALORAR LA ADHERENCIA A LA DIETA MEDITERRÁNEA (niños)

1. Toma una fruta o un zumo natural todos los días.	Sí = 1 punto	☐
2. Toma una 2ª pieza de fruta todos los días.	Sí = 1 punto	☐
3. Toma verduras frescas (ensaladas) o cocinadas regularmente una vez al día.	Sí = 1 punto	☐
4. Toma verduras frescas o cocinadas de forma regular más de una vez al día.	Sí = 1 punto	☐
5. Consume pescado con regularidad (por lo menos 2-3 veces al a semana).	Sí = 1 punto	☐
6. Acude una vez o más a la semana a un centro de comida rápida (fast food) tipo hamburguesería.	Sí = -1 punto	☐
7. Le gustan las legumbres y las toma más de 1 vez a la semana.	Sí = 1 punto	☐
8. Toma pasta o arroz casi a diario (5 dias o más a la semana)	Sí = 1 punto	☐
9. Desayuna un cereal o derivado (pan, etc)	Sí = 1 punto	☐
10. Toma frutos secos con regularidad (al menos 2-3 veces a la semana	Sí = 1 punto	☐
11. Se utiliza aceite de oliva en casa	Sí = 1 punto	☐
12. Desayuna	Sí = -1 punto	☐
13. Desayuna un lácteo (yogurt, leche, etc).	Sí = 1 punto	☐
14. Desayuna bollería industrial, galletas o pastelitos.	Sí = 1 punto	☐
15. Toma 2 yogures y/o 40 g queso cada día.	Sí = 1 punto	☐
16. Toma golosinas y/o caramelos varias veces al día	Sí = 1 punto	☐
**PUNTUACION TOTAL:**		☐☐

CUESTIONARIO PARA VALORAR LA ADHERENCIA A LA DIETA MEDITERRANEA (adultos)

1. ¿Usa usted el aceite de oliva como principal grasa para cocinar?	Sí= 1 punto	☐
2. ¿Cuánto aceite de oliva consume en total al día (incluyendo el usado para freír, comidas fuera de casa, ensaladas, etc.)?	4 o más cucharadas = 1 punto	☐
3. ¿Cuántas raciones de verdura u hortalizas consume al día? (las guarniciones o acompañamientos = 1/2 ración) 1 ración = 200g.	2 o más (al menos una de ellas en ensalada o crudas) = 1 punto	☐
4. ¿Cuántas piezas de fruta (incluyendo zumo natural) consume al día?	3 o más al día= 1 punto	☐
5. ¿Cuántas raciones de carnes rojas, hamburguesas, salchichas o embutidos consume al día? (ración: 100 - 150 g)	Menos de 1 al día= 1 punto	☐
6. ¿Cuántas raciones de mantequilla, margarina o nata consume al día? (porción individual: 12 g)	Menos de 1 al día= 1 punto	☐
7. ¿Cuántas bebidas carbonatadas y/o azucaradas (refrescos, colas, tónicas, bitter) consume al día?	Menos de 1 al día= 1 punto	☐
8. ¿Bebe usted vino? ¿Cuánto consume a la semana?	7 o más vasos a la semana = 1	☐
9. ¿Cuántas raciones de legumbres consume a la semana? (1 plato o ración de 150 g)	3 o más a la semana= 1 punto	☐
10. ¿Cuántas raciones de pescado-mariscos consume a la semana? (1 plato pieza o ración: 100 - 150 de pescado o 4-5 piezas o 200 g de marisco)	3 o más a la semana= 1 punto	☐
11. ¿Cuántas veces consume repostería comercial (no casera) como galletas, flanes, dulce o pasteles a la semana?	Menos de 2 a la semana = 1 punto	☐
12. ¿Cuántas veces consume frutos secos a la semana? (ración 30 g)	3 o más a la semana= 1 punto	☐
13. ¿Consume usted preferentemente carne de pollo, pavo o conejo en vez de ternera, cerdo, hamburguesas o salchichas? (carne de pollo: 1 pieza o ración de 100 - 150 g)	Sí= 1 punto	☐
14. ¿Cuántas veces a la semana consume los vegetales cocinados, la pasta, arroz u otros platos aderezados con salsa de tomate, ajo, cebolla o puerro elaborada a fuego lento con aceite de oliva (sofrito)?	2 o más a la semana= 1 punto	☐
**PUNTUACION TOTAL:**		☐☐

ALIMENTANDO EL CAMBIO

Centro	Participante	Fecha actual
		Día / Mes / Año
|__|__|	|__|__|__|	|__|__|__|__|__|__|

Cuestionario para padres/tutores

Queridos padres/tutores. Ustedes estan participando….

¿Cómo rellenar este cuestionario?

En las siguientes secciones te preguntamos primero cosas sobre ti y luego sobre tu hijo/a.

- Por favor, rellena el cuestionario con un **bolígrafo azul o negro**.
- Marca con una X en la casilla de respuesta. Marca solo una respuesta por pregunta aunque haya respuesta múltiple. Esto se indicará en la misma pregunta.
- En algunas preguntas te pediremos que escribas tu respuesta.

EJEMPLOS:

¿Qué distancia hay entre la guardería/colegio y tu casa?

❑1 Hasta 500 metros
❑2 Desde 500 metros a <1 kilómetro
X3 Desde 1 kilómetro a <2 kilómetros
❑4 Desde 2 kilómetros a <3 kilómetros
❑5 Desde 3 kilómetros a <4 kilómetros
❑6 4 kilómetros o más

¿Cuántas horas hace tu hijo/a deporte en un club deportivo a la semana?

|_**0**_|_**2**_| horas |_**3**_|_**5**_| minutos

Actividad física

En la siguiente sección del cuestionario te preguntaremos por tus hábitos de actividad física y de tu hijo/a.
Es importante recordar que no hay respuestas correctas o incorrectas. Contesta lo que se corresponda a tu situación.
En las siguientes preguntas, cuando decimos ACTIVIDAD FÍSICA, incluyendo el practicar un deporte o hacer ejercicio nos referimos a: Actividades que hacen al individuo respirar con más intensidad o sudar, por ejemplo, andar, ir en bicicleta, deportes de equipo como el fútbol y actividades organizadas como la natación.

PREGUNTAS ACERCA DE PADRE / MADRE O TUTOR LEGAL

Piensa en el tiempo que estuviste andando los últimos 7 días. Esto incluye andar en el trabajo y en casa, andar cuando vas de un sitio a otro, y cualquier otra actividad que hayas hecho, aunque sea en el tiempo libre, deporte, ejercicio o diversión.

Durante **los últimos 7 días**, ¿cuántos días anduviste por lo menos 10 minutos seguidos?

Sí anduve |___| días a la semana

No anduve ningún día |___|

¿Cuánto tiempo anduviste uno de estos días?

|___|___| horas al día y |___|___| minutos al día

ALIMENTANDO EL CAMBIO

Centro	Participante	Fecha actual Día Mes Año

Piensa en todas las actividades moderadas que tú realizaste en los últimos 7 días. Nos referimos a actividades que requieren esfuerzo físico moderado y que te hacen respirar de forma más intensa de lo normal. Piensa sólo en aquellas actividades físicas que hiciste por lo menos durante 10 minutos seguidos.

Durante los **últimos 7 días**, ¿Cuántos días realizaste actividad física **moderada** como llevar peso, ir en bicicleta a ritmo normal, o jugaste a tenis?

Sí1 |___| días a la semana

NO2 hice actividad física moderada |___|

¿Cuánto tiempo estuviste realizando actividad física moderada en uno de estos días?

|___|___| horas al día y |___|___| minutos al día

Piensa en todas las actividades vigorosas que tú realizaste en los últimos 7 días. Nos referimos a actividades que requieres esfuerzo físico intenso y que te hacen respirar de forma mucho más intensa de lo normal. Piensa sólo en aquellas actividades que realizaste por lo menos durante 10 minutos seguidos.

Durante **los últimos 7 días**, ¿cuántos días realizaste actividad física **vigorosa** como levantar mucho peso, cavar en tierra, aerobic, o ir en bicicleta rápido?

Sí1 |___| días a la semana

NO2 hice actividad física intensa |___|

¿Cuánto tiempo estuviste realizando actividad física intensa uno de estos días?

|___|___| horas al día y |___|___| minutos al día

Piensa en el tiempo que estuviste sentado los días de semana durante los últimos 7 días. Incluye el tiempo en el trabajo, en casa y durante el tiempo de ocio. Esto quiere decir tiempo sentado en un escritorio, visitando amigos, leyendo o sentado o tumbado viendo la TV.

Durante los últimos 7 días, ¿cuánto tiempo estuviste sentado entre semana?

|___|___| horas al día y |___|___| minutos al día

PREGUNTAS ACERCA DE TU HIJO/A

En las siguientes preguntas, cuando decimos ACTIVIDAD FÍSICA **, incluyendo el practicar un deporte o ejercicio**, nos referimos a:

Actividades que realiza tu hijo/a antes o después del colegio y que le hacen respirar de forma más intensa de lo normal o sudar.

Ejemplos de actividad física son: andar, ir en bicicleta, jugar en el parque, deportes de equipo como fútbol y actividades organizadas como nadar o clases de baile

ALIMENTANDO EL CAMBIO

Centro		Participante			Fecha actual Día		Mes		Año	

¿Pertenece tu hijo/a a un club deportivo?

❑1 Sí
❑2 No

¿Cuánto tiempo está tu hijo/a practicando deporte en el club deportivo a la semana?

|___|___| horas |___|___| minutos

¿Qué tipo de deporte practica tu hijo/a en el club deportivo?

Por favor, señala todas las que correspondan

❑1 Fútbol
❑2 Artes marciales (por ejemplo, judo)
❑3 Natación
❑4 Gimnasia rítmica
❑5 Otras, por favor, especificar: ____

¿Cómo va tu hijo/a al colegio y cómo vuelve, y cuánto tiempo dura cada viaje?

	Viaje de ida	Tiempo (minutos)	Viaje de vuelta	Tiempo (minutos)
Andando				
En bicicleta (por sí sólo)				
En bicicleta (llevado por tí)				
En bus escolar y/o otro transporte público				
En coche/motocicleta				
Otro, por favor, especificar :				

Actividades sedentarias

En la siguiente sección del cuestionario te preguntaremos por tus hábitos en actividades sedentarias y los de tu hijo/a. Es importante recordar que no hay respuestas correctas o incorrectas. Contesta lo que se corresponda a tu situación.
Cuando decimos actividades SEDENTARIAS, nos referimos a todas las actividades en las que estás sentado o tumbado, como ver la TV y/o DVD, utilizar el ordenador, dibujar o leer libros.
Respecto a las actividades frente a las pantallas, nos referimos al tiempo que inviertes en actividades como ver la TV/DVD/Video, juegos electrónicos y uso del ordenador con fines de ocio, etc.

PREGUNTAS ACERCA DE PADRE / MADRE O TUTOR LEGAL

¿Cuántas horas al día ves tú habitualmente la TV (incluyendo DVD y videos) en tu tiempo libre? (por favor, marca una casilla para los días entre semana y una casilla para los días de fin de semana)

Entre semana (media de los días)	Fines de semana (media de los días)
❑1 Nunca	❑1 Nunca
❑2 Menos de 30 minutos/día	❑2 Menos de 30 minutos/día
❑3 30 minutos a <1 hr/día	❑3 30 minutos a <1 hr/día
❑4 1- 2 hrs/ día	❑4 1- 2 hrs/ día
❑5 3-4 hrs/ día	❑5 3-4 hrs/ día
❑6 5-6 hrs/ día	❑6 5-6 hrs/ día
❑7 7-8 hrs/ día	❑7 7-8 hrs/ día
❑8 8 hrs/ día	❑8 8 hrs/ día
❑9 Más de 8 hrs/ día	❑9 Más de 8 hrs/ día
❑10 No lo sé	❑10 No lo sé

ALIMENTANDO EL CAMBIO

Centro		Participante			Fecha actual Día		Mes		Año	

¿Cuántas horas al día estás tú habitualmente utilizando el ordenador para actividades como chatear online, internet, correo electrónico o jugando, también con consolas (por ejemplo, Playstation, Xbox, GameCube) durante tu tiempo de ocio? <u>Por favor no cuentes las horas de ordenador para actividades relacionadas con el trabajo, incluso si las realizas en casa</u>

Entre semana (media de los días)	Fines de semana (media de los días)
❑1 Nunca	❑1 Nunca
❑2 Menos de 30 minutos/día	❑2 Menos de 30 minutos/día
❑3 30 minutos a <1 hr/día	❑3 30 minutos a <1 hr/día
❑4 1- 2 hrs/ día	❑4 1- 2 hrs/ día
❑5 3-4 hrs/ día	❑5 3-4 hrs/ día
❑6 5-6 hrs/ día	❑6 5-6 hrs/ día
❑7 7-8 hrs/ día	❑7 7-8 hrs/ día
❑8 8 hrs/ día	❑8 8 hrs/ día
❑9 Más de 8 hrs/ día	❑9 Más de 8 hrs/ día
❑10 No lo sé	❑10 No lo sé

¿Cuánto tiempo estás <u>tú o tu pareja</u> viendo la TV, DVD/video junto a tu hijo/a?

❑1 Nunca
❑2 Menos de una vez a la semana
❑3 Una vez a la semana
❑4 2-4 días a la semana
❑5 5-6 días a la semana
❑6 Todos los días, una vez al día
❑7 Todos los días, más de una vez al día

¿Tienes conexión a internet disponible en tu casa?

❑1 Sí
❑2 No

¿Tiene tu hijo/a los siguientes aparatos en su habitación?

	Sí	No
TV		
DVD		
Consola (por ejemplo, Play Station)		
Ordenador		

¿Cuántas horas duermes habitualmente por la noche? (por favor, marca una casilla para los días entre semana y otra casilla para los días de fin de semana)

Días de la semana **(media por noche)**	Días de fin de semana **(media por noche)**
❑1 Menos de 6 horas	❑1 Menos de 6 horas
❑2 6-7 horas	❑2 6-7 horas
❑3 8-9 horas	❑3 8-9 horas
❑4 10-11 horas	❑4 10-11 horas
❑5 12-13 horas	❑5 12-13 horas
❑6 14 horas	❑6 14 horas
❑7 Más de 14 horas	❑7 Más de 14 horas
❑8 No lo sé	❑8 No lo sé

ALIMENTANDO EL CAMBIO

Centro	Participante	Fecha actual Día	Mes	Año

PREGUNTAS ACERCA DE TU HIJO/A

Cuando decimos actividades SEDENTARIAS, nos referimos a todas las actividades en las que estás sentado o tumbado, como ver la TV y/o DVD, utilizar el ordenador, dibujar o leer libros. Respecto a las actividades frente a las pantallas, nos referimos al tiempo que inviertes en actividades como ver la TV/DVD/Video, juegos electrónicos y uso del ordenador con fines de ocio, etc.

¿Cuántas horas al día VE TU HIJO/A habitualmente la TV (incluyendo DVD y videos) in Su tiempo libre? (por favor, marca una casilla para los días entre semana y una casilla para los días de fin de semana)

Entre semana (media de los días)	Fines de semana (media de los días)
❑1 Nunca	❑1 Nunca
❑2 Menos de 30 minutos/día	❑2 Menos de 30 minutos/día
❑3 30 minutos a <1 hr/día	❑3 30 minutos a <1 hr/día
❑4 1- 2 hrs/ día	❑4 1- 2 hrs/ día
❑5 3-4 hrs/ día	❑5 3-4 hrs/ día
❑6 5-6 hrs/ día	❑6 5-6 hrs/ día
❑7 7-8 hrs/ día	❑7 7-8 hrs/ día
❑8 8 hrs/ día	❑8 8 hrs/ día
❑9 Más de 8 hrs/ día	❑9 Más de 8 hrs/ día
❑10 No lo sé	❑10 No lo sé

¿Cuántas horas al día está tu hijo/a habitualmente utilizando el ordenador para jugar, también con consolas (por ejemplo, Playstation, Xbox, GameCube) durante su tiempo de ocio?

Entre semana (media de los días)	Fines de semana (media de los días)
❑1 Nunca	❑1 Nunca
❑2 Menos de 30 minutos/día	❑2 Menos de 30 minutos/día
❑3 30 minutos a <1 hr/día	❑3 30 minutos a <1 hr/día
❑4 1- 2 hrs/ día	❑4 1- 2 hrs/ día
❑5 3-4 hrs/ día	❑5 3-4 hrs/ día
❑6 5-6 hrs/ día	❑6 5-6 hrs/ día
❑7 7-8 hrs/ día	❑7 7-8 hrs/ día
❑8 8 hrs/ día	❑8 8 hrs/ día
❑9 Más de 8 hrs/ día	❑9 Más de 8 hrs/ día
❑10 No lo sé	❑10 No lo sé

¿Cuántas horas al día juega tu hijo/a de manera pasiva (mirando, leyendo libros, jugando con muñecas, a coches, pintando, construcción) durante su tiempo de ocio?

Entre semana (media de los días)	Fines de semana (media de los días)
❑1 Nunca	❑1 Nunca
❑2 Menos de 30 minutos/día	❑2 Menos de 30 minutos/día
❑3 30 minutos a <1 hr/día	❑3 30 minutos a <1 hr/día
❑4 1- 2 hrs/ día	❑4 1- 2 hrs/ día
❑5 3-4 hrs/ día	❑5 3-4 hrs/ día
❑6 5-6 hrs/ día	❑6 5-6 hrs/ día

❑7 7-8 hrs/ día	❑7 7-8 hrs/ día
❑8 8 hrs/ día	❑8 8 hrs/ día
❑9 Más de 8 hrs/ día	❑9 Más de 8 hrs/ día
❑10 No lo sé	❑10 No lo sé

¿Cuántas horas duerme habitualmente por la noche tu hijo/a? (por favor, marca una casilla para los días entre semana y otra casilla para los días de fin de semana)

Días de la semana **(media por noche)**	Días de fin de semana **(media por noche)**
❑1 Menos de 6 horas	❑1 Menos de 6 horas
❑2 6-7 horas	❑2 6-7 horas
❑3 8-9 horas	❑3 8-9 horas
❑4 10-11 horas	❑4 10-11 horas
❑5 12-13 horas	❑5 12-13 horas
❑6 14 horas	❑6 14 horas
❑7 Más de 14 horas	❑7 Más de 14 horas
❑8 No lo sé	❑8 No lo sé

Pensando en el número de veces y duración de las siestas que tu hijo/a habitualmente hace; por favor, indica el TIEMPO TOTAL DEL DÍA HACIENDO SIESTAS (por favor, marca una casilla para los días entre semana y otra casilla para los días de fin de semana)

Días de la semana **(media por día de la semana)**	Días de fin de semana **(media por día de fin de semana)**
❑1 Mi hijo/a no hace siesta los días de la semana	❑1 Mi hijo/a no hace siesta los días de fin de semana
❑2 Menos de 1 hora	❑2 Menos de 1 hora
❑3 1-2 horas	❑3 1-2 horas
❑4 3-4 horas	❑4 3-4 horas
❑5 5-6 horas	❑5 5-6 horas
❑6 7-8 horas	❑6 7-8 horas
❑7 9 o más horas	❑7 9 o más horas
❑8 No lo sé	❑8 No lo sé

¡MUCHAS GRACIAS POR RELLENAR ESTE CUESTIONARIO!

Alimentando el Cambio

Centro | Participante | Fecha actual (Día / Mes / Año)

☐☐ ☐☐☐ | ☐☐ ☐☐ ☐☐

CUESTIONARIO GENERAL

Instrucciones

El cuestionario contiene respuestas y afirmaciones que deben de ser marcadas con una cruz. Por favor, marque solamente una opción, a no ser que se indique lo contrario
Si se le pide escribir, utilice la línea que aparece para contestar
Para preguntas referentes a cantidades o fechas, utilice las casillas que aquí se indican
Sáltese una pregunta solamente si aparece lo siguiente: "→ → →Por favor, continuar en …"
Si quisiera corregir alguna de las respuestas, por favor, tache la respuesta incorrecta y escriba la correcta algo más arriba / al lado
Si quisiera corregir una respuesta marcada con una cruz, por favor, tache completamente la respuesta y marque la deseada

Información General

1. ¿Cuál es su relación con el niño/a seleccionado/a?

☐ Padre/madre biológico/a ☐ Padre/madre adoptivo/a ☐ Padrastro o madrastra

☐ Padre/madre en un hogar de acogida ☐ Otro, por favor especifique: ______________________________

2. Usted es: ☐ Varón ☐ Mujer

3. ¿Cuál es fecha de nacimiento de su hijo/a? (dd/mm/aaaa) ☐☐/☐☐/☐☐☐☐

4. ¿Cuál es la fecha de nacimiento, altura y peso de los padres con los que vive el/la niño/a?

	MADRE		PADRE
(dd/mm/aaaa)	☐☐/☐☐/☐☐☐☐	(dd/mm/aaaa)	☐☐/☐☐/☐☐☐☐
Altura (cm)	☐☐☐,☐	Altura (cm)	☐☐☐,☐
Peso (kg)	☐☐☐,☐	Peso (kg)	☐☐☐,☐

5. ¿Con quién vive el niño/a la mayor parte del tiempo? Por favor, marque la respuesta que corresponda.

☐ Con sus padres

☐ Con su madre ☐ Con su madre y su nueva pareja ☐ Con su padre ☐ Con su padre y su nueva pareja

☐ La mitad del tiempo con su madre y la otra mitad con su padre ☐ Con sus abuelos u otros familiares

☐ Con padres de acogida o padres adoptivos ☐ En una institución, Ej. Orfanato ☐ Otro lugar, especificar: ________________

6. ¿Cuántas personas viven de forma permanente en el hogar donde el niño/a habitualmente reside?

Número de personas (adultos y niños): ☐☐ Número de personas menores a 18 años: ☐☐

Información acerca de su hijo/a

7. ¿Ha sido diagnosticado su hijo/a alguna vez de alguna de las siguientes enfermedades?
En caso afirmativo, indicar si la enfermedad sigue presente y la edad de aparición/primer diagnóstico

	**¿Todavía presenta la enfermedad?**	**¿A qué edad fue diagnosticada?**
☐ Alergias	☐ Sí ☐ No	☐☐ años
☐ Asma	☐ Sí ☐ No	☐☐ años
☐ Problemas ortopédicos crónicos, de huesos o de articulaciones	☐ Sí ☐ No	☐☐ años
☐ Enfermedad reumática crónica	☐ Sí ☐ No	☐☐ años
☐ Problemas dermatológicos importantes	☐ Sí ☐ No	☐☐ años
☐ Anemia	☐ Sí ☐ No	☐☐ años
☐ Diabetes mellitus	☐ Sí ☐ No	☐☐ años
☐ Síndrome metabólico	☐ Sí ☐ No	☐☐ años
☐ Obesidad	☐ Sí ☐ No	☐☐ años

Alimentando el Cambio

Centro		Participante			Fecha actual					
					Día		Mes		Año	

☐ Enfermedades musculoesqueléticas ☐ Sí ☐ No ☐☐ años

☐ Trastorno por Déficit de Atención con Hiperactividad (TDAH) ☐ Sí ☐ No ☐☐ años

☐ Problemas de crecimiento o de peso corporal. ☐ Sí ☐ No ☐☐ años
Por favor, especificar: ________________________

☐ Otras, por favor, especificar: __________________________ ☐ Sí ☐ No ☐☐ años

Hábito de desayuno de su hijo/a

8. En una semana normal, ¿con que frecuencia desayuna su hijo/a?

☐ Nunca ☐ Algunos días (1-3 días) ☐ La mayoría de los días (4-6 días) ☐ Todos los días ☐ Sólo el fin de semana

9. ¿Qué desayuna habitualmente su hijo/a?

☐ Nada no suele desayunar
☐ Leche
☐ Batidos Lácteos
☐ Yogur, queso u otros lácteos
☐ Café, chocolate, cacao
☐ Pan, tostadas
☐ Galletas
☐ Cereales de desayuno azucarados
☐ Cereales de desayuno no azucarados
☐ Bollería
☐ Fruta Fresca
☐ Zumo natural exprimido
☐ Zumo envasado
☐ Otros alimentos

Estilo de vida familiar, salud y bienestar de su hijo/a

10. En caso de necesidad real, ¿con cuántas personas (incluyendo a su familia) realmente puede contar usted?

☐ Nadie ☐ 1 persona ☐ 2 ó 3 personas ☐ Más de 3 personas

24. Bienestar emocional. ***Durante la última semana, mi hijo/a….***

… se estuvo riendo y se divirtió mucho.	☐ No del todo	☐ Casi nunca	☐ A veces	☐ A menudo	☐ Todo el tiempo
… no se sintió bien para hacer nada	☐ No del todo	☐ Casi nunca	☐ A veces	☐ A menudo	☐ Todo el tiempo
… se sintió solo/a.	☐ No del todo	☐ Casi nunca	☐ A veces	☐ A menudo	☐ Todo el tiempo
… se sintió inseguro/a y estaba ansioso/a.	☐ No del todo	☐ Casi nunca	☐ A veces	☐ A menudo	☐ Todo el tiempo

25. Autoestima. ***Durante la última semana, mi hijo/a…***

… estuvo orgulloso/a de sí mismo/a.	☐ No del todo	☐ Casi nunca	☐ A veces	☐ A menudo	☐ Todo el tiempo
… se sintió en la cima del mundo	☐ No del todo	☐ Casi nunca	☐ A veces	☐ A menudo	☐ Todo el tiempo
… se sintió bien consigo mismo/a.	☐ No del todo	☐ Casi nunca	☐ A veces	☐ A menudo	☐ Todo el tiempo
… tuvo buenas ideas.	☐ No del todo	☐ Casi nunca	☐ A veces	☐ A menudo	☐ Todo el tiempo

26. Familia. ***Durante la última semana, mi hijo/a…***

… se sintió a gusto con nosotros como padres.	☐ No del todo	☐ Casi nunca	☐ A veces	☐ A menudo	☐ Todo el tiempo
… estuvo a gusto en casa.	☐ No del todo	☐ Casi nunca	☐ A veces	☐ A menudo	☐ Todo el tiempo
… discutimos en casa.	☐ No del todo	☐ Casi nunca	☐ A veces	☐ A menudo	☐ Todo el tiempo
… se sintió sobreprotegido/a por nosotros.	☐ No del todo	☐ Casi nunca	☐ A veces	☐ A menudo	☐ Todo el tiempo

Alimentando el Cambio

Centro	Participante	Fecha actual: Día	Mes	Año

Información socio-demográfica de la familia

11. ¿Tiene su hijo/a su propia habitación? □ Sí □ No

12. ¿Cuántos coches tiene su familia?

□ No disponemos de coche □ 1 coche □ 2 coches □ 3 o más coches

13. ¿Cuántos ordenadores tiene su familia?

□ No disponemos de ordenador □ 1 ordenador □ 2 ordenadores □ 3 o más ordenadores

14. ¿Tiene conexión a internet en su casa? □ Sí □ No

15. ¿Nació su hijo/a en España? □ Sí □ No, por favor especificar: ___________________

16. ¿Nació la madre del niño/a en España? □ Sí □ No, por favor especificar: ___________________

17. ¿Nació el padre del niño/a en España? □ Sí □ No, por favor especificar: ___________________

18. ¿En qué lengua habla normalmente en casa con su hijo/a?

□ Castellano, catalán, valenciano, euskera, gallego.

□ Otro idioma, por favor especifique: _____________________

19. ¿Cuál es el nivel más alto de educación escolar de los padres del niño/a?

**Por favor, marcar solamente uno por persona.**	***Madre***	***Padre***
Primaria /EGB	❍1	❍1
Secundaria /ESO	❍2	❍2
Formación profesional	❍3	❍3
Ciclos formativos de grado superior	❍4	❍4
Bachillerato/ BUP/COU	❍5	❍5
Sin graduación (todavía)	❍6	❍6
Otros/desconocido	❍7	❍7

20. ¿Cuál es el nivel más alto de cualificación profesional que tienen los padres?

**Por favor, marcar solamente uno por persona.**	***Madre***	***Padre***
Formación profesional	❍1	❍1
Ciclos formativos de grado superior	❍2	❍2
Diplomatura universitaria/ingeniería técnica	❍3	❍3
Licenciatura/ graduado /ingeniería superior	❍4	❍4
Doctorado	❍5	❍5
No formado (todavía)	❍6	❍6
Desconocido/otros	❍7	❍7

21. ¿Cuál de los siguientes enunciados describe mejor el estado ocupacional actual de los padres del niño/a?

**Por favor, marcar solamente uno por persona.**	***Madre***	***Padre***
Trabajo a tiempo completo (30 horas o más a la semana)	❍1	❍1
Trabajo a tiempo parcial (menos de 30 horas a la semana)	❍2	❍2
Estudio o voy a la universidad	❍3	❍3
No tengo trabajo remunerado (ej. labores del hogar)	❍4	❍4
Retirado (también jubilación anticipada)	❍5	❍5
Baja temporal de la empresa (ej. baja por maternidad o paternidad)	❍6	❍6
Desempleado en la actualidad, recibiendo el paro	❍7	❍7
Desempleado en la actualidad, sin recibir el paro	❍8	❍8
En asistencia pública (asistencia social)	❍9	❍9
Otro, por favor especifique: _________________________________	❍10	❍10

22. ¿En qué posición laboral están actualmente ocupados los padres del niño/a?

Si usted o su cónyuge/pareja ya no están ocupados o actualmente no están ocupados, por favor, indique la última posición laboral.

	Madre	*Padre*
**Obrero**		
Obrero no cualificado	❍1	❍1
Obrero semi-cualificado	❍2	❍2
Obrero cualificado, artesano	❍3	❍3
Maestro artesano, capataz	❍4	❍4
**Patrón o autónomo (incluyendo la ayuda de miembros de la familia)**		
Agricultor y/o ganadero autónomo	❍1	❍1
Autónomo, trabajador por cuenta propia	❍2	❍2
Patrón con hasta 9 empleados	❍3	❍3
Patrón con 10 o más empleados	❍4	❍4
Ayudo a algún miembro de la familia	❍5	❍5
**Empleado**		
Empleado (ej. dependiente, recepcionista, oficinista)	❍1	❍1
Empleado cualificado (ej. auxiliar contable, auxiliar dental)	❍2	❍2
Empleado altamente cualificado o con funciones de gestión (ej. científico, jefe de departamento)	❍3	❍3
Empleado con extensas funciones ejecutivas (ej. director, director general, junta directiva)	❍4	❍4
**Funcionario público**		
Categoría A	❍1	❍1
Categoría B	❍2	❍2
Categoría C	❍3	❍3
Categoría D	❍4	❍4
Categoría E	❍5	❍5
**No trabajo**	❍6	❍6

¡Gracias por contestar a las preguntas!

Por favor, compruebe una vez más que ha rellenado el cuestionario en su totalidad.

Por favor, anote la fecha en la que terminó de rellenar el cuestionario: |___|___| Día |___|___| Mes|___|___|___|___| Año
